# A STUDY ON THE COMPARISON OF STRESS INDEX OF DEMENTED OLDER ADULTS AND THE PAIN OF THE MAIN CAREGIVER

**DOI:** 10.1093/geroni/igz038.412

**Published:** 2019-11-08

**Authors:** Sujin Kim, Eunhee Cho, Hwang Sinwoo, Eunji Kwon

**Affiliations:** 1 Yonsei University, Seoul, Korea, Republic of; 2 Professor, College of Nursing, Yonsei University, Seoul, Korea, Republic of

## Abstract

The purpose of this study was to compare the stress and fatigue index between the demented older adults and the non-demented older adults and to identify the correlation between the behavioral psychological symptoms of the demented older adults and the pain of the main caregiver. A total of 100 participants(80 demented older adults and 20 non-demented older adults) were selected. The demented older adults, who visited hospital neurology as an outpatient, were paired up with the caregivers who provided the care for the demented older adults. The non-demented older adults who had normal cognitive function without limitation in a daily life and scored MMSE of 24 or higher were selected. The stress and fatigue index were measured by using an autonomic nervous system analyzer that measures heart rate variability(HRV), and structured questionnaires were used to examine behavioral psychological symptoms. The collected data were analyzed by using Independent t-test and Pearson’s correlations analysis. The result of the analysis showed that average score of the demented older adults had higher stress index than the control group(non-demented older adults), which was statistically significant(t=3.350, p=.001). In addition, the result indicated the statistically significant positive correlation between the behavioral psychological symptoms of demented older adults and the level of pain of main caregivers (r=0.89, p=<.001). The results of this study suggest that managing both the physical and psychological aspects of the stress in the main caregivers is necessary, as well as the development of stress management program for the demented older adults.

